# Sociodemographic and Clinical Predictors of Self-Management among People with Poorly Controlled Type 1 and Type 2 Diabetes: The Role of Illness Perceptions and Self-Efficacy

**DOI:** 10.1155/2016/6708164

**Published:** 2015-12-01

**Authors:** Abdul-Razak Abubakari, Rosanna Cousins, Cecil Thomas, Dushyant Sharma, Ebrahim K. Naderali

**Affiliations:** ^1^School of Health and Life Sciences, Glasgow Caledonian University London, London E1 6PX, UK; ^2^Liverpool Hope University, Hope Park, Liverpool L16 9JD, UK; ^3^Diabetes and Endocrinology Department, Aintree University Teaching Hospital, Liverpool L9 7AL, UK; ^4^Diabetes and Endocrinology Department, Royal Liverpool University Hospital, Liverpool L7 8XP, UK

## Abstract

Self-management is critical if people with diabetes are to minimise their risk of macrovascular and microvascular complications, yet adherence to self-management recommendations is suboptimal. Understanding the predictors of optimal diabetes self-management in specific populations is needed to inform effective interventions. This study investigated the role of demographic and clinical characteristics, illness perceptions, and self-efficacy in explaining adherence to self-management recommendations among people with poorly controlled diabetes in North West of England. Illness perceptions and self-efficacy data were collected using validated questionnaires and clinical data were obtained from hospital records. Correlations were used to investigate bivariate relationships between independent variables and self-management, and multiple regression techniques were used to determine demographic and psychosocial predictors of self-management. Various demographic and clinical characteristics were associated with adherence to self-management recommendations. In particular, employment status explained 11% of the variation in adherence to foot care whilst diabetes treatment category explained 9% of exercise and 21% of the variations in SMBG recommendations. Also, 22% and 8% of the variations in overall self-management were explained by illness perceptions and self-efficacy beliefs, respectively. Illness perceptions and self-efficacy beliefs of people with poorly controlled diabetes are important predictors of their self-management behaviours and could potentially guide effective interventions.

## 1. Introduction

Diabetes is a complex chronic condition with serious physical, psychological, and clinical complications for individuals affected [1]. Irrespective of the type of diabetes, appropriate self-management is critical if individuals with the condition are to minimise their risk of diabetes complications and ensure improved health outcomes overall. Key areas of the diabetes self-management regime include significant behavioural and lifestyle changes such as meal and dietary planning, daily regulation of physical activity, appropriate use of recommended medication, and, where applicable, monitoring and interpretation of blood glucose and use of its results to inform decisions such as adjusting medications, diet, and physical activity levels [2]. There is evidence that adherence to supportive but often complex self-management plans in diabetes is suboptimal [3, 4] which raises questions about potential predictors of effective self-management, which have particular importance for people whose diabetes presents as poorly controlled.

In addition to the drastic behavioural and lifestyle changes required following diagnosis with diabetes, uncertainties about the future and, indeed, thoughts and/or experiences of the acute and chronic complications associated with the condition often lead to severe coping and other psychosocial problems for individuals affected. Thus, promoting effective self-management requires that patients are equipped with a repertoire of relevant knowledge and skills through appropriate self-management education and support systems [2, 5]. Specifically, individuals with diabetes need a clear understanding of the tasks involved in self-management, a practical appreciation of how to perform each self-management task on daily basis, some of which could be complex, and the ability to determine when and under what circumstances to undertake a particular self-management task, as well as decision-making and problem-solving skills [6, 7]. Even with these skills, adherence to self-management recommendations is influenced by several other factors including (but not limited to) personal and sociodemographic characteristics, individual's own perceptions and expectations about the given illness, and their perceived confidence in relation to whether or not they are able to perform the given self-management task (concept of self-efficacy) [8–10].

Self-efficacy is a cognitive theory that was put forward in the 1970s by Bandura [11]. The concept asserts that individuals' level of confidence in relation to their ability to perform a given task such as a specific health behaviour is an important determinant of whether or not they initiate and engage in that behaviour. Because of its potential to influence the desired health outcomes, the concept of self-efficacy is of great interest to researchers, health providers, and promoters in search of theoretical frameworks to anchor and guide policy and practice. Particularly, improvements in self-efficacy of patients have been used as a mechanism to enhance behaviour change and improve adherence to chronic disease self-management recommendations, including those for diabetes [6, 12, 13]. Indeed, self-management interventions developed with self-efficacy as the underpinning theoretical framework have shown promise, albeit inconclusive. For instance, Lorig and Holman [6] observed that self-efficacy on its own significantly influences the health status of people with long-term conditions. Specifically they found that self-efficacy levels at baseline as well as changes in self-efficacy achieved through self-management intervention significantly predicted health status. Recently, others have also reported modest to strong relationships between self-efficacy and self-management behaviours among adults with type 2 diabetes [14, 15] and adolescents with type 1 diabetes [9, 16]. In spite of the plethora of evidence on the relationship between self-efficacy and self-management, the majority of studies have been conducted in the general diabetes population, regardless of patient demographics and diabetes control outcomes. Thus, it is not clear if such a relationship will exist in a sample exclusively drawn from a population with poorly controlled diabetes.

In addition to self-efficacy, another cognitive framework useful for explaining health-related behaviour choices of individuals and perhaps populations is the illness representation model. This concept is based on the common-sense model of illness representation which evolved from the work of Howard Leventhal and colleagues' investigating impact of fear messages on individuals' inclination to perform recommended health behaviour [17]. They observed that a health threatening stimulus provoked, simultaneously, the search for both emotional and cognitive representations of the health threat among their study participants. Leventhal noted that the parallel processing of the cognitive and emotional representations of the threat served to generate strategies and coping plans with which to eliminate the threat. Much of research on this framework has been on patient samples; however, in principle, a health threat (stimulus) could result from experiencing symptoms of an illness, being diagnosed with an illness, or being potentially at risk of illness. Illness representations could emerge from three types of information: “lay” knowledge already possessed by an individual, authoritative information obtained from external sources (e.g., doctor and book), and personal experience, whether current or previous, including outcomes of that experience. It is contended that an individual's representations of an illness are based on their personal perceptions and/or experiences of the condition or other illness and should not necessarily be expected to conform to existing medical facts about the given illness [18, 19].

Research has reliably shown that an individual represents an illness along five cognitive dimensions [17]: identity, the label and symptoms associated with the illness; consequences, the individual's perception of how the illness might affect their lives and any likely outcome; Control, the individual's perception of the level of control or influence they have over the course of the illness, including its cure or treatment; timeline, the length of time individuals perceive their illness will last (short-term or long-term); and Cause, individuals' beliefs about what caused the illness. The emotional representation component indicates patients' attitude or state of mind in response to the diagnosis or health threat (e.g., fear, anxiety, or distress). Illness representations have been found to significantly influence individual's lifestyle and behaviour choices, and the concept has subsequently been employed as an effective mechanism for improving behaviour change and other health outcomes [20–22]. Among people with diabetes, illness representation has been reported to predict adherence to recommended self-management behaviours, as well as objective clinical outcomes, particularly glycaemic control levels [23–26]. However, what is not clear is whether the concept of illness representation is universal in terms of the demographic and clinical profile of the patient population studied. This is important if effective packages to support coping and self-management of diabetes are to be put forward by clinicians.

This study examined whether patient characteristics, patients' diabetes specific self-efficacy, or patients' illness representations would significantly influence self-management behaviours of predominantly poorly controlled type 1 and type 2 diabetes (from this point, type 1 and type 2 diabetes will be referred to as diabetes, unless otherwise specified) patients managed in acute trusts. We specifically aimed at addressing 2 research questions. First, we sought to investigate which sociodemographic and disease characteristics are likely to influence diabetes specific self-efficacy, illness representation, and self-management among individuals with poorly controlled diabetes. Then we determined whether self-efficacy and illness representations influence the degree of adherence to self-management recommendations among the study population.

## 2. Materials and Methods

### 2.1. Study Participants and Procedures

This study was part of a larger study that investigated the influence of work-related factors on diabetes self-management. Thus, names and addresses of potentially working adults (aged 25–65 years) with diabetes were obtained from databases of two NHS hospital trusts in the North West of England. Individuals who did not have a record of severe mental (such as severe depression) or cognitive disorder (such as dementia) and could provide consent were contacted by post and invited to participate in the study. Study packs containing invitation letter, consent form, participant information sheet, stamped self-addressed return envelope, and study questionnaires were sent to potential participants. The invitation letter asked patients to read the information sheet and if they agree to participate in the study, sign the consent forms, complete the questionnaires, and return them to the researchers in the stamped envelope provided. After three weeks from the date of postage, reminder letters and another pack of questionnaires were sent to patients who did not respond. Participant recruitment took place between July 2013 and November 2013.

Relevant data were collected using a demographic and disease characteristics questionnaire, the brief Illness Perceptions Questionnaire (IPQ) [27], the Summary of Diabetes Self-care Activities (SDSCA) questionnaire [28], and the Perceived Diabetes Self-Management Scale (PDSMS) [29].

Recent (last 6 months, if not last 12 months) data on glycaemic control (HbA1c), diabetes complication status (microvascular), and comorbidities were obtained from participants' medical records. Ethical approval was granted by the North East-Newcastle & North Tyneside committee 1.

### 2.2. Study Questionnaires

The brief IPQ [27] is a nine-item summarised and quick to administer version of the full illness perception questionnaires [30, 31] used for assessing individuals' cognitive and emotional representations of an illness such as diabetes. The first eight items of the questionnaire examine patient's perceptions of the timelines, consequences, identity (symptom load), coherence (or understanding), and emotions, each scored on a scale of 0 to 10. The ninth item asks patients to rank in order of importance the 3 factors that they believe to have caused their illness (in this case, diabetes). Overall, high scores on the brief IPQ indicate a more threatening or serious view of the illness, whereas low scores reflect more benign view. Psychometric properties of the instrument have been evaluated using a wide range of patient populations including people with myocardial infarction, asthma, and diabetes. Six-week test-retest reliability for individual components of the measure, as determined by Pearson correlation coefficient, ranged between 0.42 and 0.75 and items of the brief IPQ correlated sufficiently with equivalent component items of the full IPQ-R (ranging from 0.32 to 0.63), indicating good concurrent validity. Further, various elements of the brief IPQ have been found to significantly predict a range of health outcomes including attendance at rehabilitation classes, time of return to work, and quality of life among MI patients [27].

The SDSCA is a self-report measure and asks patients to indicate how many of the last seven days they fulfilled dietary, exercise, self-monitoring of blood glucose (SMBG), and foot care recommendations as advised by their diabetes health care team (scored on a scale of 0 days–7 days). The revised SDSCA used in this study consists of 11 carefully selected core items which demonstrated sound psychometric properties (internal consistency, predictive validity, and no ceiling/floor effect) from seven previously published studies [28]. Of the 11 items of the SDSCA, four are for diet, two are for exercise, two are for SMBG, two are for foot care, and one is for smoking. In consonance with the number of days in a week, each item (except smoking) is scored on a scale of zero (participant has not performed the task in the last seven days) to seven (participant has performed the task every day in the past seven days). The item for smoking is binary (Yes/No response) and asks whether participants smoked cigarette during the past seven days. In this study, the scores for specific dimension of self-management, diet, exercise, SMBG, and foot care, were obtained from the average scores of all relevant items combined. Scores for overall adherence to self-management (overall self-management) were computed by taking the average of diet, exercise, SMBG, and foot care. The smoking item was not included in computing the dimension-specific or the overall self-management scores.

The PDSMS [29] is an 8-item likert-type questionnaire which was developed from the generic Perceived Medical-Condition Self-Management Scale [32] for use with both type 1 and type 2 diabetes populations. If fully completed, total scores for the PDSMS range from 8 to 40 with high scores indicating greater confidence in the individual's ability to self-manage their diabetes. Psychometric evaluation analyses showed reasonable correlations between responses to the PDSMS and SDSCA and demographic characteristics, demonstrating sufficient evidence of construct validity. The PDSMS also showed good internal consistency (Cronbach's alpha = 0.83) among the mixed type 1 and type 2 diabetes patient sample.

### 2.3. Statistical Methods

Data analyses were conducted using the Statistical Package for Social Sciences (SPSS version 20, IBM Corp.). Initial analyses were performed to investigate normality and other assumptions of parametric statistical tests where required. Bivariate correlations (Pearson's correlation coefficient (*r*) for continuous variables, Spearman's rho (*P*) for ordinal and continuous variables that were not normally distributed, or point biserial (*r*_pb_) for dichotomous variables; see Table 1) were produced to examine univariate relationships between dependent and independent variables. Two methods of multiple regressions were used. First, stepwise regressions were used to determine which sociodemographic characteristics best predict psychosocial dependent variables (illness perceptions and self-efficacy) and adherence to self-management recommendations. Finally, the enter method was used to determine the extent to which illness perceptions or self-efficacy predicts adherence to self-management recommendations in the study population. Independent variables were entered irrespective of the strength of the relationship in the bivariate correlations.

Based on relevant parameters of the main study, a minimum of 340 participants were required to achieve a precision of 0.05 at 95% confidence interval, assuming 1 in 3 diabetes patients in the working-age range was in employment. However, a minimum sample size of 128 is sufficient for the analysis presented in this paper (see details in Section 3.2.2).

People with diabetes are not a homogenous group and some questions of the SDSCA may not be relevant for some specific groups. For instance, SMBG may not be recommended for some patients, particularly individuals on oral hypoglycaemic agents. Thus, prior to completing the SDSCA, participants were asked to indicate (Yes/No/Not applicable) whether they have ever been advised by their diabetes healthcare team to perform any of the recommended self-management tasks since diagnosis. Responses to this preliminary question were then used to adjust for all analysis involving the SDSCA.

## 3. Results and Discussions

### 3.1. Results

#### 3.1.1. Demographic and Disease Characteristics of Study Participants

A hundred and twenty-three individuals with diabetes (51% type 2) participated in the study. Just over half (51%) of participants were male and nearly all participants (96%) considered their ethnicity as white British. About a quarter of participants (24%) had university level educational qualification and most (80%) were employed or self-employed. On average, participants were 50 years old (mean age: 50.24; SD = 10.84), had been diagnosed with diabetes for 16 years (mean duration since diagnosis = 15.97 years; SD = 10.62), and were on average obese as indicted by the mean BMI = 31.76 (SD = 6.90). As expected of an exclusively poorly controlled sample, average percentage HbA1c was 44.39 (SD = 14.22). Prevalence of diabetes related comorbidity and complications were also high in the studied population with over 80% of participants having blood pressure levels considered either prehypertensive or hypertensive. Also, half of participants had diabetic retinopathy, slightly more than one in five (21%) had diabetic neuropathy, and 11% were diagnosed with nephropathy.

#### 3.1.2. Associations between Sociodemographic and Disease Characteristic Variables and Psychosocial Dependent Variables

Bivariate correlations between demographic/disease characteristics and individual components of illness representations showed scores of significant relationships. Notably, high educational attainment was associated with perception of greater personal control (*P* = 0.21, *p* value < 0.05) and less concern about their illness (*P* = −0.22, *p* value < 0.05). Participants with type 1 diabetes tended to perceive their diabetes as long-term (*r* = −0.26, *p* value < 0.01) and had perceptions of greater personal control (*r* = −0.29, *p* value < 0.01), greater treatment control (*r* = −0.31, *p* value < 0.01), and greater understanding or illness coherence (*r* = −0.21, *p* value < 0.05). As a surrogate marker of illness severity, being on a more complex treatment regimen was associated with experiencing greater number of symptoms (*P* = 0.35, *p* value < 0.001). Longer duration since diagnosis was significantly correlated with perceiving diabetes as long-term condition (*r* = 0.28, *p* value < 0.01) and perceptions of greater personal control (*r* = 0.21, *p* value < 0.05), treatment control (*r* = 0.20, *p* value < 0.05), and greater understanding or coherence about diabetes (*r* = 0.30, *p* value < 0.01). High BMI was associated with greater worries (concern) about diabetes (*P* = 0.22, *p* value < 0.05), presence of neuropathy was associated with perception of less treatment effectiveness (*r* = −0.29, *p* value < 0.01), and presence of nephropathy was associated with perceptions of greater consequences (*r* = 0.20, *p* value < 0.05) as a result of diabetes.

In terms of overall illness perception score, the univariate relationships (see Table 1 column 2) indicate that, compared to the unemployed/retired, participants who were employed had more threatening representations about their diabetes (see interpretation of brief IPQ in Section 2.2). Also having type 2 diabetes, being on a more complex treatment regimen, having higher BMI, and having neuropathy were associated with a more threatening view of diabetes.

As shown in Table 2, a raft of significant relationships between demographic/disease characteristics and illness perceptions were observed in the multivariate regression analyses. Demographic/disease characteristics explained 12% of the variations in consequences, 56% of timeline, 38% of personal control, 27% of treatment control, 17% of identity (symptom load), 15% of concern, 14% of illness coherence, and 9% of emotional representations among study participants. A key demographic variable which predicted variations in specific components of illness perceptions was educational achievement. Compared with the lowest educational attainment (primary/secondary education), college/sixth form graduates were less likely to represent their diabetes as long-term (*B* = −0.50; 95% CI, −0.77–−0.24; *p* value ≤ 0.001) and graduates from university/graduate were more likely to report greater personal control of their diabetes (*B* = 1.30; 95% CI, 0.29–2.30; *p* value ≤ 0.01), whereas graduates from polytechnic were more likely to perceive their diabetes with greater number of symptoms (*B* = −2.44; 95% CI, −4.72–−0.15; *p* value ≤ 0.05). In relation to disease or clinical variables, treatment regimen, duration since diagnosis, microvascular complication status, type of diabetes, BMI, and percentage HbA1c significantly contributed to a range of specific illness representations. Diabetes treatment category emerged as a significant predictor for most specific components of illness perceptions. Specifically, there were significant variations in consequences (*B* = 1.34; 95% CI, 0.20–2.48; *p* value ≤ 0.05), timeline (*B* = −0.67; 95% CI, −1.04–−0.30; *p* value ≤ 0.001), personal control (*B* = 1.56; 95% CI, 0.12–3.00; *p* value ≤ 0.05), identity (number of symptoms experienced) (*B* = 1.86; 95% CI, 0.67–3.05; *p* value ≤ 0.01), and concern (*B* = −1.86; 95% CI, −2.81–−0.90 *p* value ≤ 0.001) between participants on one hypoglycaemic tablet compared to people on more complex or advanced treatment regimens (≥2 hypoglycaemic tablets, insulin, or diabetic tablets and insulin). Longer duration since diagnosis was associated with longer timeline perceptions (*B* = 0.02; 95% CI, 0.01–0.03; *p* value ≤ 0.01), greater understanding (coherence) about diabetes (*B* = 0.06; 95% CI, 0.02–0.11; *p* value ≤ 0.01), and lower negative emotional response (*B* = −0.09; 95% CI, −0.15–−0.03; *p* value ≤ 0.01).

Employment status, diabetes type, diabetes treatment category, BMI, and neuropathy status significantly contributed to the overall illness perception scores. The multivariate relationships between demographic/disease characteristics and overall illness perception as shown in Table 2 (row (ix)) indicate that diabetes treatment category (1 diabetic tablet versus diabetic tablets and insulin), nephropathy status, duration since diagnosis, percentage HbA1c, and BMI significantly contributed to variations in illness representation in the study sample. Together, these variables explained 34% of the variation in overall illness representation among the study participants.

Diabetes type and duration since diagnosis significantly correlated with self-efficacy scores in univariate analyses (Table 1). Having type 1 diabetes and longer duration since diagnosis was associated with higher confidence in patients' ability to self-manage their diabetes. In the regression analysis (see part (2) of Table 2), neuropathy status, duration since diagnosis, and percentage HbA1c each contributed significantly in predicting patients' perceived confidence (self-efficacy). The three variables together explained 23% of the variation in self-efficacy among the study participants.

#### 3.1.3. Associations between Sociodemographic/Disease Characteristic Variables and Adherence to Self-Management Recommendation

A number of sociodemographic/disease characteristic variables significantly influenced participants' self-management behaviours in the univariate analysis as captured in Table 1. Older age was associated with greater adherence to foot self-management and being female was associated with greater frequency of SMBG. Consistent with general expectations, participants with type 1 diabetes, those with more complex treatment regimens and longer duration since diagnosis, were also associated with higher frequency of SMBG. Further, employment status, diabetes type, percentage HbA1c, and neuropathy status were also associated with adherence to exercise recommendations. The multivariate analyses to determine which demographic/disease characteristics predict self-management behaviours showed that only diabetes treatment category and employment status significantly contributed to variations in self-management behaviours. As seen in Table 2 part (3), diabetes treatment category explained small but significant proportions of the variations in participants' adherence to exercise (9%), SMBG (21%), and overall self-management (7%) whilst employment status explained 11% of the variation in adherence to foot care recommendations.

#### 3.1.4. Associations between (a) Illness Representations and Self-Management Behaviours and (b) Self-Efficacy for Diabetes Self-Management and Self-Management Behaviours

Multivariate relationships between illness representation scores and measured diabetes self-management behaviours are shown in Table 3 part (1). The results suggest that illness representations explain significant proportions of the variations in adherence to SMBG (14%), feet care (18%), and overall self-management (22%). Specifically longer timeline representations of diabetes were associated with greater frequency of SMBG (*B* = 0.66; 95% CI, 0.07–1.25; *p* value < 0.05). Greater sense of personal control (*B* = 0.25; 95% CI, 0.04–0.46; *p* value < 0.05) and illness coherence (*B* = 0.28; 95% CI, 0.06–0.49; *p* value < 0.01) was associated with better adherence to feet care. In addition, greater perception of illness coherence (*B* = 0.19; 95% CI, 0.08–0.31; *p* value < 0.001) was associated with higher adherence to overall self-management recommendations.

Results for relationships between self-efficacy for managing diabetes and self-reported adherence to self-management recommendations (as in Table 3 part (2)) indicate that self-efficacy is a predictor of patients' adherence to diabetes self-management recommendations. This is particularly significant for overall self-management in which self-efficacy explained 8% of the variation in self-management among the participants.

### 3.2. Discussion

#### 3.2.1. Summary of Findings

This study investigated clinico-sociodemographic and psychosocial predictors of self-management behaviours among individuals with poorly controlled diabetes receiving care at two acute trusts in North West of England. In accordance with our research questions, the findings suggest that participants who were employed had more threatening representations about their diabetes compared with less economically active (unemployed/retired) participants.

Participants with adverse or advanced clinical outcomes, complex treatment regimen, being diagnosed with nephropathy, high hbA1c, and high BMI, were more likely to have threatening representations about their diabetes.

Compared to participants with type 2 diabetes, individuals with type 1 diabetes expressed higher confidence in their ability to self-manage their diabetes. Three clinical indicators contributed significantly in predicting participants' self-efficacy for diabetes self-management, duration since diagnosis, neuropathy status, and HbA1c. Participants who have been diagnosed with diabetes for a longer duration perceived greater confidence in self-managing their diabetes, whereas participants who have been diagnosed with neuropathy and those with higher HbA1c perceived lower confidence in their ability to self-manage the condition. Type of diabetes, neuropathy status, duration since diagnosis, and HbA1c were also confirmed as significant predictors of self-efficacy for self-management of diabetes in multiple regression analyses.

A range of demographic (particularly, educational attainment) and disease (treatment category, duration since diagnosis, microvascular complication status, type of diabetes, BMI, and HbA1c) characteristics contributed significantly in predicting patients' representations about their diabetes.

A variety of demographic and disease characteristic variables were significantly associated with participants' self-management behaviours. In particular, treatment category significantly explained participants' degree of adherence to exercise (9%), SMBG (21%), and overall self-management (7%) recommendations.

Finally, both illness representations and self-efficacy for diabetes self-management were significant predictors of participants' self-management behaviours. Illness representations explained 14% of adherence to SMBG, 18% of adherence to foot care, and 22% of overall self-management recommendations. Self-efficacy beliefs explained 3% each of adherence to diet and exercise and 8% of overall self-management recommendations.

#### 3.2.2. Discussion of Findings

The gender distribution of the sample reflects the slight differences in the overall prevalence of diabetes in men (6.3%) and women (5.3%) in England (33) and the age distribution of the sample is consistent with the mixed sample of younger type 1 diabetes patients (mean age: 45.10; SD = 12.03) and older type 2 participants (mean age: 55.05; SD = 7.03).

The finding that illness representations predict self-management behaviours of this poorly controlled diabetes population shows that the Leventhal's common sense model of illness representation is fairly robust in explaining patients' thoughts and reflections in their attempt to cope with a health condition such as diabetes [33]. It also shows the concept is applicable to a wide range of patients with diabetes, irrespective of their demographic, cultural, or clinical profile [9, 10, 24, 25]. Similar observations have been shown in studies of other illness groups too. For example, a meta-analysis examining the significance of illness perceptions on attendance at cardiac rehabilitation following acute myocardial infarction reported small but significant effect sizes of the relationships between the two variables [21]. In relation to specific components of the illness perception schema, meta-analysis of the results found that greater perception of identity or symptom load (*r* = 0.13; *p* value = 0.004), consequences (*r* = 0.08, *p* value = 0.012), and cure/control (*r* = 0.119, *p* value < 0.001) were significantly associated with attendance at cardiac rehabilitation [21].

The fact that longer duration since diagnosis was associated with longer timeline perceptions, greater understanding (coherence) about diabetes and lower negative emotional response is encouraging and reflects a situation where patients have come to terms with the reality about their condition and therefore doing their best to comprehend and potentially confront the illness rather than allow themselves to be weighed down by negative emotions.

Although somewhat expected, the finding that self-efficacy significantly explains variations in adherence to self-management behaviours is of immense practical importance. Indeed, self-efficacy is a social cognitive concept which has behavioural underpinnings including the motivation for the individual to activate and persist on the behaviour even in the face of difficulties, albeit depending on the magnitude and strength of the efficacy expectations [11]. In part, self-efficacy is driven by an individual's expectations that behaving in a particular way will yield benefits and/or avert difficulties in the future. The motivation to initiate and sustain behaviour could also be activated through the mechanism of goal setting and attainment, self-initiated or otherwise [34].

Our findings on self-efficacy and self-management behaviours concur with the large body of literature which consistently demonstrates associations between self-efficacy and health-related behaviours. For example, in an ethnically diverse, low income population with type 2 diabetes, Sarkar and colleagues reported significant association between self-efficacy and adherence to dietary, exercise, SMBG, and foot care recommendations [35]. The associations observed in their study persisted after adjusting for relevant clinical and demographic variables. More recently, Walker and others also reported moderately significant associations between participants' adherence to diet, exercise, foot care, and SMBG among low income and minority ethnic populations with diabetes, both in univariate and multivariate (but not foot care) analyses [15]. Indeed as found in our study others [15, 35] have also shown significant associations between self-efficacy and clinical disease outcomes such as HbA1c.

Interestingly, both psychological measures (the brief IPQ and the PDSMS) identified persons with type 1 diabetes and having been diagnosed with diabetes for a longer duration to be associated with higher perceived confidence. On the contrary, presence of neuropathy and high HbA1c were associated with lower perceived confidence in managing diabetes. Unfortunately, the cross-sectional nature of this study does not permit us to make inferences about temporality of these relationships. For instance, it is unclear whether individuals are likely to become less confident after failing to bring their HbA1c under control or developing diabetic complications or vice versa. Nevertheless, this finding is useful and could guide the identification of patients who may benefit from self-efficacy related interventions.

Our findings show that sociodemographic and disease characteristics (mostly in the univariate analyses, but also a few in the multivariate analyses) influenced adherence to self-management recommendations in different ways. Exploring such variations further could be useful for targeting and tailoring self-management interventions to specific population groups [36, 37].

In terms of its implications for practice, the pivotal role of self-efficacy in predicting or acting as a catalyst for motivating the individual in the performance of a given task, including health behaviour, implies that the concept could be used to guide behaviour change and diabetes control intervention in our study population, as has been demonstrated in other populations with diabetes [12, 14]. As explained by Bandura [11] the magnitude and intensity of self-efficacy possessed by an individual could vary for different areas or aspects of behaviour; it is therefore possible to identify any deficiencies in an individual and work towards augmenting them through the provision of targeted skill training and education [38]. This approach could potentially work for poorly controlled patients such as the participants in our study.

An obvious implication of the findings in relation to illness representation is that illness representation in general could be used as a framework to guide interventions aiming at promoting appropriate self-management behaviours among individuals with poorly controlled diabetes. Particularly, interventions enhancing appropriate timeline, personal control, and illness coherence components of the illness perception schema could be useful. On the flip side, it is also possible to use the illness perception concept as a screening tool for identifying patients who, because of their illness representations, are potentially less likely to adhere to self-management recommendations and subsequently working to alter these perceptions [20, 39].

This study has some limitations which should be considered in the interpretation of its findings. First, the small sample size of the study (*n* = 123) means there is potential for type II error. For example, considering the sample size calculation based on the 50 + 8*k* (*k* = number of predictors) rule for multiple regression analysis [40], we would have required at least 128 complete responses for the multiple regression analysis between self-management and the eight components of illness representation. It is therefore likely that our analyses failed to detect some associations. Secondly, the participant information sheet for the main study explained that the study was investigating work-related factors on diabetes self-management. Invitation letters and study packs were accordingly sent to all patients in the working age range (25–65) as the hospital records did not contain this information. Indeed, some patients returned questionnaires uncompleted, some with notes explaining that they are not in employment. It would therefore be misleading to present statistics for response rates in this report. Also, data for both predictor and outcome variables were collected at a single time point (cross-sectional design). Cross-sectional studies have several limitations, including the fact that we are unable to infer causality and direction of effect in the associations reported in this study. Further, data were collected using postal questionnaires and some clinical data obtained from routine hospital records. Both postal questionnaires and routine data have their inherent limitations too. Nonetheless, the findings from this small scale study are of critical importance and further studies investigating the applicability of illness representations and self-efficacy frameworks and their practical underpinnings on the behaviour of exclusively poorly controlled individuals with diabetes are warranted.

## 4. Conclusions

The concept of self-management is about helping patients assume the day-to-day control of their illness with some support from healthcare professionals. Thus we call for more studies aimed at understanding the potential barriers and promoters of self-management and good clinical control of diabetes in predominantly poorly controlled diabetes populations such as our study sample, using psychological constructs such as those used in this study. Different forms of reaching out to patients of varying ages and clinical and demographic profiles including digital platforms which could be tailored to the circumstances of individuals may be appropriate.

## Figures and Tables

**Table 1:** Bivariate relationships between sociodemographic and disease characteristic variables and psychosocial dependent variables.

Variable	IP score	PDSMS score	Diet SMG	Exercise SMG	SMBG	Foot SMG	Overall SMG
Age	−0.03	−0.02	0.03	−0.12	−0.01	0.24^*∗*^	0.08
Sex_pb_	0.04	−0.10	0.12	0.03	0.18^*∗*^	0.02	0.16
Marital status_*ρ*_	0.16	−0.12	−0.14	−0.16	−0.07	−0.01	−0.11
Educational qualification_*ρ*_	−0.16	0.16	−0.09	0.12	−0.01	0.10	−0.02
Ethnicity	0.01	0.03	0.09	0.05	−0.05	0.22^*∗*^	0.12
Employment status_*ρ*_	0.23^*∗*^	−0.09	−0.04	−0.20^*∗*^	−0.04	0.08	0.01
Diabetes type_*ρ*_	0.19^*∗*^	−0.20^*∗*^	0.06	−0.23^*∗*^	−0.26^*∗∗*^	0.13	−0.09
Diabetes treatment type_*ρ*_	0.21^*∗*^	−0.08	0.05	−0.14	0.39^*∗∗∗*^	0.07	0.12
Duration since diagnosis	−0.18	0.19^*∗*^	−0.01	0.05	0.33^*∗∗∗*^	0.13	0.18
BMI status_*ρ*_	0.21^*∗*^	−0.13	−0.08	−0.19	−0.16	0.15	−0.10
HbA1c (%) categories_*ρ*_	−0.12	0.18	−0.18	0.22^*∗*^	0.07	0.04	0.10
BP status_pb_	0.07	−0.01	0.12	−0.01	−0.09	0.15	0.02
Retinopathy status_pb_	−0.01	0.03	−0.07	−0.04	0.11	0.07	0.07
Neuropathy status_pb_	0.24^*∗*^	−0.14	−0.11	−0.20^*∗*^	−0.08	0.13	−0.09
Nephropathy status_pb_	0.14	−0.06	0.10	0.09	−0.04	0.05	0.12
IP score			−0.16	−0.13	−0.01	0.04	−0.17
PDSMS score			−0.16	0.19^*∗*^	0.10	0.17	0.29^*∗∗*^

*ρ*, spearman's rho; pb, point biserial correlation coefficient; all other correlation coefficients are Pearson's correlation coefficients.

^*∗*^ *p* value ≤ 0.05; ^*∗∗*^ *p* value ≤ 0.01; ^*∗∗∗*^ *p* value ≤ 0.001.

IP, illness perception; PDSMS, Perceived Diabetes Self-Management Scale; SMG, self-management; SMBG, self-monitoring of blood glucose.

**Table 2:** Predicting illness perceptions, self-efficacy, and adherence to self-management recommendations from sociodemographic and disease characteristics.

Predictor variable	*B* (SE)	95% CI	*p* value	*β*	*R* ^2^
(1) *Predicting illness perceptions from demographic/disease characteristics *					
(i) Consequences and demographic/disease characteristics			<0.01^*∗∗*^		0.12
Constant	2.88 (0.41)	0.88–4.89	≤0.01^*∗∗*^		
Nephropathy status	2.54 (1.03)	0.48–4.59	0.02^*∗*^	0.254	
diabetic tablets + insulin versus 1 diabetic tablet	1.34 (0.57)	0.20–2.48	0.02^*∗*^	0.242	
HbA1c (%)	0.04 (0.02)	0.00–0.08	0.04^*∗*^	0.212	
(ii) Timeline and demographic/disease characteristics			<0.001		0.56
Constant	9.78 (0.13)	9.55–10.05	<0.001^*∗∗∗*^		
≥2 diabetic tablets versus 1 diabetic tablet	−0.67 (0.19)	−1.04–−0.30	0.001^*∗∗∗*^	−0.34	
Primary/secondary versus college/sixth form	−0.50 (0.13)	−0.77–−0.24	<0.001^*∗∗∗*^	−0.36	
Duration since diagnosis	0.02 (0.01)	0.01–0.03	<0.01^*∗∗*^	0.29	
(iii) Personal control and demographic/disease characteristics			<0.001^*∗∗∗*^		0.38
Constant	12.79 (1.16)	10.48–15.11	<0.001^*∗∗∗*^		
Diabetes type	−2.78 (0.51)	−3.78–−1.79	<0.001^*∗∗∗*^	−0.56	
HbA1c (%)	−0.07 (0.02)	−0.10–−0.04	<0.001^*∗∗∗*^	−0.39	
Primary/secondary versus university/graduate	1.30 (0.51)	0.29–2.30	0.01^*∗∗*^	0.23	
≥2 diabetic tablets versus 1 diabetic tablet	1.56 (0.73)	0.12–3.00	0.03^*∗*^	0.21	
(iv) Treatment control and demographic/disease characteristics			<0.001^*∗∗∗*^		0.27
Constant	13.00 (1.17)	10.68–15.33	<0.001^*∗∗∗*^		
Diabetes type	−1.73 (0.49)	−2.70–−0.75	0.001^*∗∗∗*^	−0.36	
HbA1c (%)	−0.05 (0.02)	−0.09–−0.02	<0.01^*∗∗*^	−0.30	
Neuropathy status	−1.47 (0.59)	−2.64–−0.30	0.01^*∗∗*^	−0.25	
(v) Identity and demographic/disease characteristics			≤0.001^*∗∗∗*^		0.17
Constant	4.63 (0.42)	3.79–5.17	<0.001^*∗∗∗*^		
diabetic tablets and insulin versus 1 diabetic tablet	1.86 (0.60)	0.67–3.05	<0.01^*∗∗*^	0.32	
Primary/secondary versus polytechnic	−2.44 (1.15)	−4.72–−0.15	0.04^*∗*^	−0.22	
(vi) Concern and demographic/disease characteristics			<0.001^*∗∗∗*^		0.15
Constant	8.22 (0.29)	7.65–8.79	<0.001^*∗∗∗*^		
insulin versus 1 diabetic tablet	−1.86 (0.48)	−2.81–−0.90	<0.001^*∗∗∗*^	−0.39	
(vii) Illness coherence and demographic/disease characteristics			<0.01^*∗∗*^		0.14
Constant	5.82 (0.50)	4.83–6.81	<0.001^*∗∗∗*^		
Duration since diagnosis	0.06 (0.02)	0.02–0.11	<0.01^*∗∗*^	0.30	
Sex	1.05 (0.46)	0.14–1.96	0.02^*∗*^	0.24	
(viii) Emotional response and demographic/disease characteristics			<0.01^*∗∗*^		0.09
Constant	6.67 (0.60)	5.48–7.86	<0.001^*∗∗∗*^		
Duration since diagnosis	−0.09	−0.15–−0.03	<0.01^*∗∗*^	−0.30	
(ix) Overall IP score and demographic/disease characteristics			<0.001^*∗∗∗*^		0.34
Constant	14.21	−3.09–31.51	0.11		
diabetic tablets and insulin versus 1 diabetic tablet	6.82 (2.22)	2.40–11.23	<0.01^*∗∗*^	0.29	
Nephropathy status	10.98 (3.87)	3.27–18.70	<0.01^*∗∗*^	2.84	
Duration since diagnosis	−0.21 (0.11)	−0.42–0.00	0.06	−0.19	
HbA1c (%)	0.27 (0.09)	0.10–0.44	<0.01^*∗∗*^	0.32	
BMI	0.48 (0.19)	0.11–0.86	0.01^*∗∗*^	0.27	
(2) *Predicting PDSMS scores from demographic/disease characteristics *			**<**0.001^*∗∗∗*^		**0.23**
Constant	31.87 (2.56)	26.77–36.98	<0.001^*∗∗∗*^		
Neuropathy status	−5.17 (1.63)	−8.42–−1.92	<0.01^*∗∗*^	−0.32	
Duration since diagnosis	0.17 (0.06)	0.05–0.29	<0.01^*∗∗*^	0.29	
HbA1c (%)	−0.14 (0.05)	−0.24–−0.03	0.01^*∗∗*^	−0.26	
(3) *Which demographic/disease characteristics best predict adherence to self-management recommendations*?					
(i) Diet/meal planning recommendations and demographic/disease characteristics					
No variable entered into the equation (no variable significantly predicted diet)					
(ii) Exercise recommendations and demographic/disease characteristics			**<**0.01^*∗∗*^		**0.09**
Constant	2.21 (0.31)	1.59–2.83	<0.001^*∗∗∗*^		
Insulin versus 1 diabetic tablet	1.47 (0.52)	0.44–2.50	0.01^*∗∗*^	0.31	
(iii) Blood testing recommendations and demographic/disease characteristics					0.21
Constant	5.17 (0.27)	4.63–5.71	<0.001^*∗∗∗*^		
≥2 diabetic tablets versus 1 diabetic tablet	−3.49 (0.75)	−4.97–−2.00	<0.001^*∗∗∗*^	−0.46	
(iv) Foot care recommendations and demographic/disease characteristics			**<**0.01^*∗∗*^		**0.11**
Constant	2.53 (0.26)	2.02–3.05	<0.001^*∗∗∗*^		
Employed versus not in work due to long-term illness/disability	2.32 (0.75)	0.82–3.81	<0.01^*∗∗∗*^	0.32	
(v) Overall SMG recommendations and demographic/disease characteristics			0.02^*∗*^		0.07
Constant	3.79 (0.15)	3.50–4.10	<0.001^*∗∗∗*^		
≥2 diabetic tablets versus 1 diabetic tablet	−0.99 (0.42)	−1.83–−0.16	0.02^*∗*^	−0.26	

^*∗*^ *p* value ≤ 0.05; ^*∗∗*^ *p* value ≤ 0.01; ^*∗∗∗*^ *p* value ≤ 0.001.

**Table 3:** Predicting adherence to recommended self-management behaviours from psychosocial variables (illness perceptions or self-management-related self-efficacy).

Predictor variable	*B* (SE)	95% CI	*p*-value	*β*	*R* ^2^
(1) *Predicting self-management from illness perceptions *					
Diet recommendations and illness perceptions			0.07^**∗**^		**0.13**
Constant	5.43 (2.05)	1.37–9.48	0.01^*∗∗*^		
Consequences	0.04 (0.08)	−0.13–0.21	0.65	0.06	
Timeline	−0.26 (0.20)	−0.64–0.13	0.19	−0.13	
Personal control	0.01 (0.08)	−0.15–0.17	0.93	0.01	
Treatment control	0.03 (0.09)	−0.15–0.17	0.73	0.04	
Identity	−0.10 (0.07)	−0.24–0.03	0.13	−0.16	
Concern	−0.06 (0.08)	−0.22–0.09	0.42	−0.09	
Understand	0.21 (0.08)	0.05–0.37	0.01^**∗∗**^	0.27	
Emotional response	0.07 (0.07)	−0.07–0.21	0.33	0.12	
Exercise recommendations and illness perceptions			**0.14**		**0.11**
Constant	4.27 (2.76)	1.21–9.75	0.13		
Consequences	0.02 (0.12)	−0.23–0.26	0.90	0.02	
Timeline	−0.36 (0.26)	−0.88–0.16	0.18	−0.13	
Personal control	−0.09 (0.12)	−0.33–0.14	0.43	−0.10	
Treatment control	0.29 (0.13)	0.02–0.56	0.03^**∗**^	0.27	
Identity	−0.16 (0.09)	−0.35–0.02	0.08	−0.19	
Concern	−0.08 (0.11)	−0.30–0.15	0.50	−0.08	
Understand	0.05 (0.11)	−0.17–0.27	0.65	0.05	
Emotional response	0.17 (0.10)	−0.03–0.36	0.09	0.22	
SMBG recommendations and illness perceptions			0.04^**∗**^		**0.14**
Constant	−4.77 (3.11)	−10.94–1.40	0.13		
Consequences	0.10 (0.13)	−0.15–0.36	0.42	0.11	
Timeline	0.66 (0.30)	0.07–1.25	0.03^**∗**^	0.20	
Personal control	−0.04 (0.13)	−0.29–0.21	0.75	−0.04	
Treatment control	0.22 (0.15)	−0.08–0.51	0.15	0.18	
Identity	0.15 (0.10)	−0.05–0.35	0.15	0.15	
Concern	−0.19 (0.12)	−0.43–0.05	0.11	−0.18	
Understand	0.15 (0.12)	−0.10–0.39	0.24	0.12	
Emotional response	0.05 (0.11)	−0.16–0.27	0.62	0.06	
Foot care recommendations and illness perceptions			0.01^*∗∗*^		**0.18**
Constant	3.52 (2.73)	−1.90–8.94	0.20		
Consequences	0.15 (0.11)	−0.07–0.38	0.17	0.18	
Timeline	−0.39 (0.26)	−0.91–0.12	0.13	−0.14	
Personal control	0.25 (0.11)	0.04–0.46	0.02^**∗**^	0.25	
Treatment control	−0.17 (0.13)	−0.42–0.07	0.17	−0.17	
Identity	0.09 (0.09)	−0.08–0.26	0.31	0.10	
Concern	−0.09 (0.10)	−0.29–0.12	0.41	−0.09	
Understand	0.28 (0.11)	0.06–0.49	≤0.01^*∗∗*^	0.26	
Emotional response	0.06 (0.09)	−0.12–0.25	0.50	0.08	
Overall self-management recommendations and illness perceptions			0.003^*∗∗*^		**0.22**
Constant	1.93 (1.44)	−0.92–4.79	0.18		
Consequences	0.03 (0.07)	−0.10–0.16	0.66	0.06	
Timeline	−0.06 (0.14)	−0.33–0.21	0.65	−0.04	
Personal control	0.02 (0.06)	−0.10–0.15	0.71	0.04	
Treatment control	0.10 (0.07)	−0.05–0.24	0.18	0.16	
Identity	−0.05 (0.05)	−0.15–0.05	0.30	−0.11	
Concern	−0.07 (0.06)	−0.18–0.05	0.30	−0.13	
Understand	0.19 (0.06)	0.08–0.31	≤0.001^*∗∗∗*^	0.33	
Emotional response	0.10 (0.05)	−0.001–0.20	0.05^**∗**^	0.23	
(2) *Predicting self-management recommendations from self-efficacy (PDSMS score) *					
Diet recommendations and PDSMS score					**0.03**
Constant	3.50 (0.58)	2.35–4.65	<0.001^*∗∗∗*^		
Diabetes self-efficacy	0.04 (0.02)	−0.001–0.08	0.05^**∗**^	0.18	
Exercise recommendations and PDSMS score					**0.03**
Constant	0.88 (0.93)	−0.96–2.73	0.34		
Diabetes self-efficacy	0.06 (0.03)	−0.00–0.13	0.05^**∗**^	0.19	
SMBG recommendations and PDSMS score					**0.01**
Constant	3.46 (1.06)	1.36–5.56	0.001^*∗∗∗*^		
Diabetes self-efficacy	0.04 (0.04)	−0.04–0.12	0.29	0.10	
Foot care recommendations and PDSMS score					**0.03**
Constant	1.28 (0.94)	−0.58–3.14	0.18		
Diabetes self-efficacy	0.06 (0.03)	−0.01–0.13	0.07	0.17	
Overall SMG recommendations and PDSMS score					**0.08**
Constant	2.23 (0.48)	1.27–3.18	<0.001^*∗∗∗*^		
Diabetes self-efficacy	0.05 (0.02)	0.02–0.09	<0.01^*∗∗*^	0.29	

^*∗*^ *p* value ≤ 0.05; ^*∗∗*^ *p* value ≤ 0.01; ^*∗∗∗*^ *p* value ≤ 0.001.
